# Intracranial complete response for non‐small‐cell lung cancer patient with negative PD‐L1 expression of the lung using nivolumab

**DOI:** 10.1002/cnr2.1460

**Published:** 2021-05-27

**Authors:** Rui Kitadai, Yoshitaka Zenke, Tsunekazu Hishima, Yukio Hosomi

**Affiliations:** ^1^ Department of Thoracic Oncology and Respiratory Medicine Tokyo Metropolitan Cancer and Infectious Diseases Center Komagome Hospital Bunkyo Japan; ^2^ Department of Thoracic Oncology National Cancer Center Hospital East Kashiwa Japan; ^3^ Department of Pathology Tokyo Metropolitan Cancer and Infectious Diseases Center Komagome Hospital Bunkyo Japan

**Keywords:** central nervous system metastases, nivolumab, non‐small‐cell lung cancer, programmed cell death 1 ligand 1 negative

## Abstract

**Background:**

Nivolumab has shown promising results against non‐small‐cell lung cancer (NSCLC). However, its efficacy to treat central nervous system (CNS) metastases, specifically among programmed cell death 1 ligand 1 (PD‐L1)‐negative patients, remains unclear.

**Case:**

A 66‐year‐old woman was diagnosed with adenocarcinoma stage II and underwent a left lower lobectomy. The histopathological evaluation revealed stage IVA with pleural dissemination. The patient did not harbor an epidermal growth factor receptor (EGFR) mutation or anaplastic lymphoma kinase (ALK) rearrangement, and PD‐L1 expression of the surgical specimen using 22C3 assay was 0%. Single brain metastasis was detected, and carboplatin and nab‐paclitaxel were administered. After three cycles, asymptomatic multiple brain metastases were identified, and the patient was treated with nivolumab as second‐line chemotherapy. Six months later, MRI revealed an intracranial complete response (CR). Nivolumab was discontinued after 23 cycles due to immune‐related adverse events (irAEs) of grade 2 rash. However, its effects were sustained for 13 months after discontinuation. We were unable to evaluate the PD‐L1 expression of brain metastases, which may show heterogeneity.

**Conclusion:**

This case demonstrates that nivolumab effectively treated a patient with negative PD‐L1 expression of the lung harboring CNS metastases.

## 1 INTRODUCTION

Central nervous system (CNS) metastasis is a common complication of advanced non‐small‐cell lung cancer (NSCLC) and has been found in 24%–44% of patients.^1^ The prognosis of patients with CNS metastasis is poor.^2^ Local strategies such as surgery and radiotherapy have been included in standard care. The efficacy of chemotherapy is limited due to the blood–brain barrier. However, clinical trials have documented intracranial responses to targeted therapies, including epidermal growth factor receptor (EGFR) mutation and anaplastic lymphoma kinase (ALK) tyrosine kinase inhibitors.

Nivolumab is a human immunoglobulin G4 anti‐programmed cell death 1 (PD‐1) antibody, which was approved as monotherapy for the second‐line treatment of advanced NSCLC. Based on clinical trials, the effects of nivolumab last longer than that of docetaxel.^3^, ^4^ Its efficacy depends on the PD‐L1 expression in tumor cells.^4^ However, few reports on the efficacy of nivolumab against CNS metastases, especially in patients with negative PD‐L1 expression. We presented the case of a PD‐L1‐negative NSCLC patient who achieved CR of brain metastases after nivolumab monotherapy.

## 2 CASE

**FIGURE 1:**
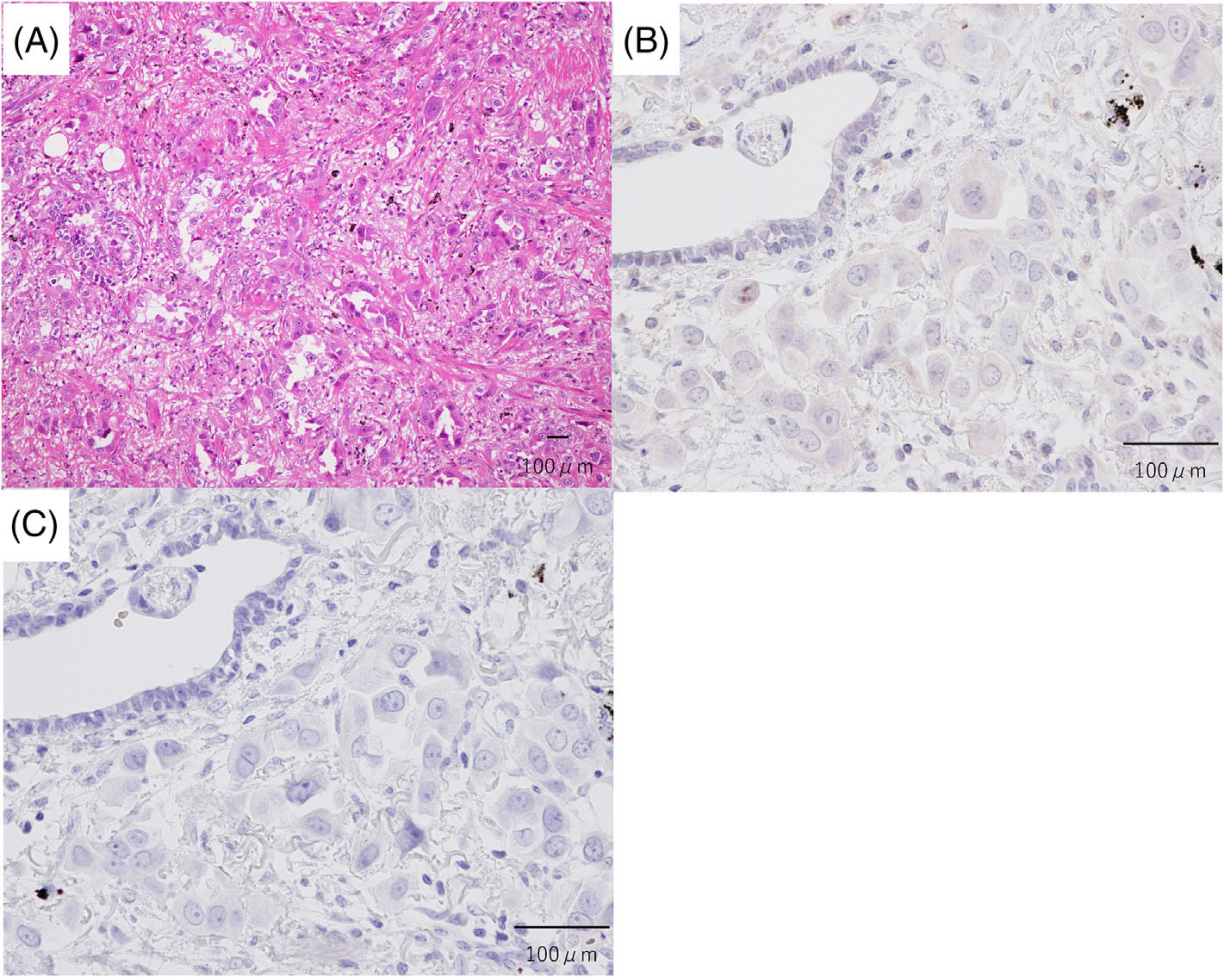
(A) A histological examination revealed that the tumor was an adenocarcinoma (hematoxylin and eosin staining). (B) Immunohistochemistry for PD‐L1 (22C3 assay) showed a tumor proportion score of 0%. (C) A negative control for the immunohistochemistry

**FIGURE 2:**
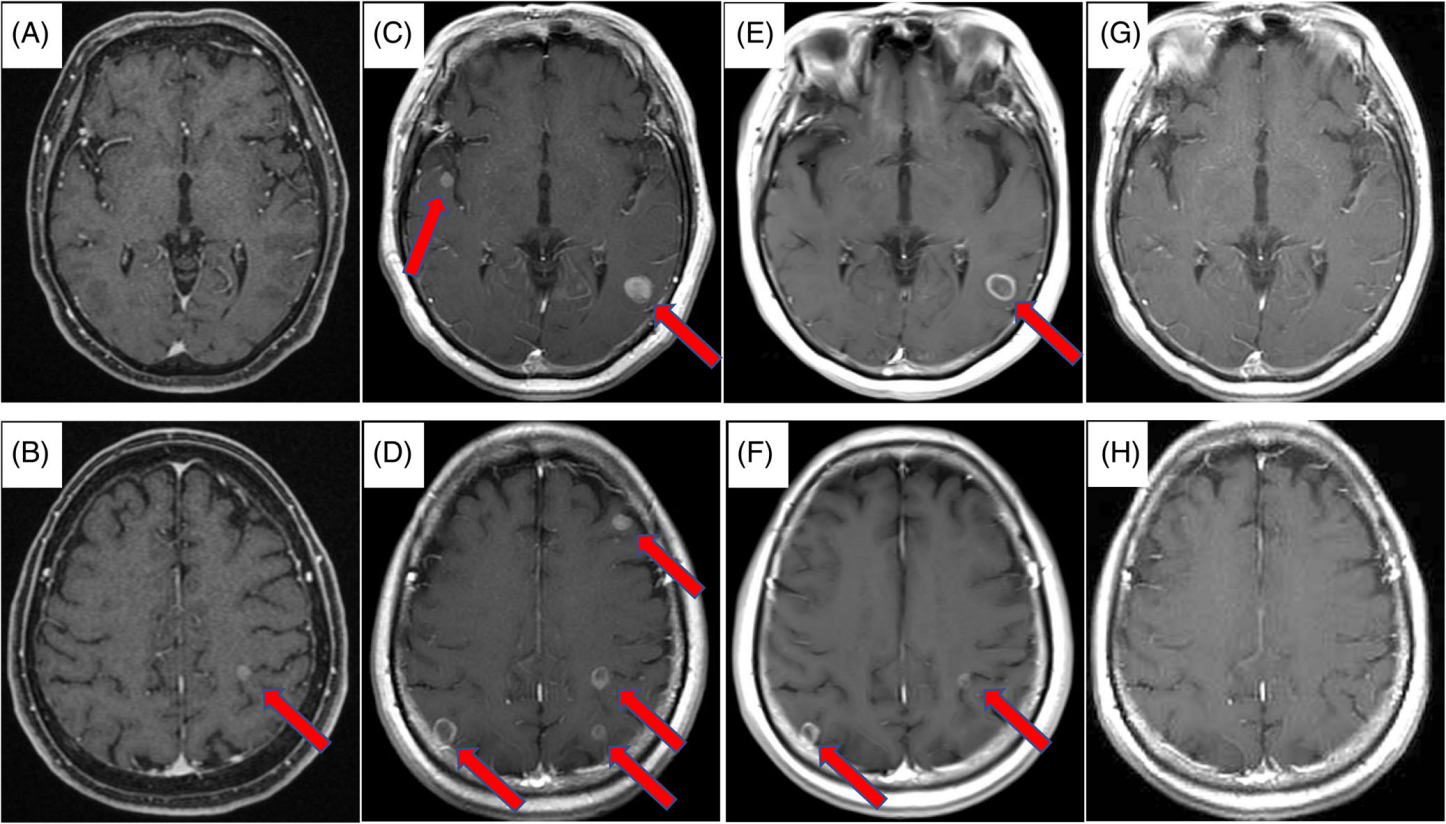
(A, B) A single brain metastasis was detected before chemotherapy. (C, D) Multiple brain metastases were identified after three cycles of carboplatin and nab‐paclitaxel treatment. (E, F) Tumor shrinkage of the brain metastases was observed 1 month after the initiation of nivolumab. (G, H) A complete response of brain metastases was observed after 6 months of nivolumab treatment

**FIGURE 3:**
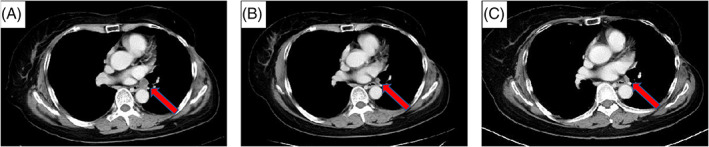
(A) The mediastinal lymph node, which is the target lesion, before carboplatin and nab‐paclitaxel treatment. The targeted lesion before (B) and after (C) nivolumab treatment

A 66‐year‐old woman presented with a chest X‐ray abnormality, suggesting adenocarcinoma cT2aN1M0 Stage IIA of the left lower lobe. The patient had a 52.5 pack‐year smoking history. The Eastern Cooperative Oncology Group performance status was 0. She underwent a left lower lobectomy in March 2016, and the histopathological evaluation revealed an adenocarcinoma pT2aN2M1a Stage IVA (pleural dissemination), without EGFR mutation and ALK translocation (Figure 1(A)). One month later, a brain MRI scan revealed a 6 mm enhancing left parietal lobe mass with no neurologic symptoms. Carboplatin (area under the curve, 5 mg/ml/min on day 1, every 3 weeks) and nab‐paclitaxel (100 mg/m^2^ weekly) were administered. However, she developed grade 2 chemotherapy‐induced peripheral neuropathy, and the treatment was discontinued after three cycles. Asymptomatic multiple brain metastases were identified, and the patient was treated with nivolumab (3 mg/kg, day 1, every 2 weeks) as second‐line therapy. One month later, the MRI scan showed tumor shrinkage of the brain metastases. The tumor PD‐L1 expression of the primary lung tumor was 0% based on the 22C3 assay (Figure 1(B, C)). Six months later, MRI showed complete response (CR) for brain metastases (Figure 2), and a computed tomography (CT) scan showed stable disease of the primary lesion (Figure 3). Eleven months later, nivolumab was discontinued after the patient had developed a grade 2 rash. However, the patient has sustained intracranial CR for 13 months without nivolumab treatment. Next‐generation sequencing (NGS) was performed afterward using the original lung tumor, and TP53 K321 alteration and RB1 E19 alteration were detected. Due to the tissue condition, the tumor mutation burden (TMB) could not be determined.

## 3 DISCUSSION

Patients with brain metastases have often been excluded from clinical trials. Recent trials have shown the promising efficacy of immune checkpoint inhibitors (ICIs) against brain metastases. A summary of studies reporting the efficacy of ICI monotherapy for brain metastases in patients with NSCLC is shown in Table 1.

**TABLE 1:** Summary of the efficacy of ICI monotherapy for brain metastases in patients with NSCLC

Author	Study design	ICI	Number of patients	PD‐L1 status (%)	Brain metastases RR (%)	Number of intracranial CR	Median PFS (months)	Median OS (months)
Hendriks et al.^5^	Retrospective	PD‐1 inhibitor or PD‐L1 inhibitor	255	Positive 20%; negative 13%; unknown 67%	27.3	NR	1.7	8.6
Kim et al.^6^	Retrospective	Pembrolizumab or nivolumab	18	Positive16.7%; negative 11.1%; unknown 72.2%	11.1	0	NR	NR
Song et al.^7^	Retrospective	Pembrolizumab or nivolumab or atezolizumab	5	NR	40	0	NR	8.6
Bjornhart et al.^8^	Retrospective	Pembrolizumab or nivolumab	21	< 1% 5%; 1%–50% 14%; ≥50% 57%; unknown 24	13	1	NR	8.2
Gauvain et al.^9^	Retrospective	Nivolumab	43 (30 were assessable)	< 1% 11.6%; 1 ≥ 11.6%; unknown 76.8%	9	NR	3.9	not reached
Cortnovis et al.^10^	Retrospective	Nivolumab	37	NR	19	1	4.9	5.8
Dudnik et al.^11^	Retrospective	Nivolumab	5	NR	40	1	NR	NR
Goldberg et al.^12^	Phase II	Pembrolizumab	Cohort 1:37 Cohort 2:5	Cohort1: ≥1% Cohort 2: <1% or not evaluable	Cohort 1:29.7 Cohort 2:0	4	Cohort 1:1.9	Cohort 1:9.9

Abbreviations: BM, brain metastases; CR, complete response; ICI, immune checkpoint inhibitor; NR, not reported; NSCLC, non‐small ‐cell lung cancer; OS, overall survival; PD‐L1, programmed cell death 1 ligand 1; PFS, progression‐free survival; RR, response rate.

The PD‐L1 status is a predictor of nivolumab efficacy in nonsquamous NSCLC. Some studies have reported discordance in the expression of PD‐L1 between primary lesions and metastatic sites in lung cancer. A study of 73 cases showed that the discordance in the expression of PD‐L1 between primary lung tumors and their corresponding metastatic brain lesions was 14%.^13^ In this case, we were unable to evaluate the PD‐L1 expression of brain metastases. The brain metastases likely had a higher PD‐L1 expression than the primary lesion, resulting in a positive response to treatment. Response to nivolumab has been reported in some PD‐L1‐negative cases.^3^ Other factors such as tumor mutation burden, tumor microenvironment with tumor‐infiltrating lymphocytes, and DNA mismatch repair also affected the efficacy.^14^

NGS has been used to detect driver mutations and TMB that can be used as biomarkers of treatment response. Molecular‐targeted drugs are approved for NSCLC with driver mutations such as EGFR, ALK, ROS1, BRAF, MET, and NTRK, showing remarkable efficacy. Some drugs show intracranial efficacy, which could be an option for patients with CNS metastases. Patients with TMB‐high NSCLC who received ICI therapy showed significantly longer PFS and OS than those with TMB‐low NSCLC,^15^ which suggests that TMB may be a predictive biomarker for patients treated with ICI. The association between TP53 mutations and longer survival has been reported in patients with NSCLC who underwent ICI treatment.^16^ Patients with TP53 mutations exhibited a higher response rate than those without a mutation.^16^ In our case, the patient harbored a TP53 mutation, which may be associated with her complete response. On the other hand, a retrospective study reported a lack of response to immunotherapy in RB1‐mutated advanced and recurrent NSCLC.^17^ Further investigation is needed for the relationship between the effects of RB1 mutation and ICI.

The mechanism behind the effects of PD‐1 inhibitors on brain metastases remains unclear. Preclinical models have shown that ICI activates T‐cell trafficking across the blood–brain barrier without directly acting on the tumor.^18^ This suggested that ICIs were effective against brain metastases.

Unlike cytotoxic chemotherapy, PD‐1 inhibitors elicit a long‐term response, which persists even after treatment discontinuation. The 5‐year survival was 13.4% according to pooled efficacy and safety data from the phase III CheckMate‐017 and CheckMate‐057 trials.^19^ Our patient had a stable response against brain metastases lasting 25 months.

To our knowledge, this was the first case report to show complete response to nivolumab against brain metastases in patients with PD‐L1‐negative NSCLC. Further research is needed to better understand the mechanism and identify biomarkers that can better predict the efficacy of nivolumab.

## CONFLICT OF INTEREST

Yoshitaka Zenke reports grants from AstraZeneca, MSD K. K., and Merck; personal fees from AstraZeneca, Boehringer‐Ingelheim, Chugai Pharmaceutical Co. Ltd., Eli Lily Co. Ltd., MSD K. K., Ono Pharmaceutical Co., and Bristol‐Meyers Squibb. The other authors have no conflicts of interest to disclose. All authors were involved in manuscript drafting/revising and approved the final manuscript.

## AUTHORS' CONTRIBUTIONS

All authors had full access to the data in the study and take responsibility for the integrity of the data and the accuracy of the data analysis. *Conceptualization*, R.K., Y.Z., Y.H.; *Formal Analysis*, R.K.; *Resources*, T.H., Y.Z.; *Writing ‐ Original Draft*, R.K.; *Writing ‐ Review & Editing*, Y.Z., H.Y.; *Supervision*, Y.Z.

## ETHICAL STATEMENT

Informed consent was obtained to publish this report.

## Data Availability

The data that support the findings of this article are available on request from the corresponding author.
